# The Dioxin receptor modulates Caveolin-1 mobilization during directional migration: role of cholesterol

**DOI:** 10.1186/s12964-014-0057-7

**Published:** 2014-09-21

**Authors:** Javier Rey-Barroso, Alberto Alvarez-Barrientos, Eva Rico-Leo, María Contador-Troca, José M Carvajal-Gonzalez, Asier Echarri, Miguel A del Pozo, Pedro M Fernandez-Salguero

**Affiliations:** Departamento de Bioquímica y Biología Molecular, Facultad de Ciencias, Universidad de Extremadura, 06071 Badajoz, Spain; Servicio de Técnicas Aplicadas a las Biociencias, Universidad de Extremadura, 06071 Badajoz, Spain; Department of Developmental and Regenerative Biology, Mount Sinai School of Medicine, 10029 New York, USA; Departamento de Biología Vascular e Inflamación, Centro Nacional de Investigaciones Cardiovasculares (CNIC), 28029 Madrid, Spain

**Keywords:** Dioxin receptor, Caveolin-1, Membrane microdomains, Endocytosis, Cholesterol

## Abstract

**Background:**

Adhesion and migration are relevant physiological functions that must be regulated by the cell under both normal and pathological conditions. The dioxin receptor (AhR) has emerged as a transcription factor regulating both processes in mesenchymal, epithelial and endothelial cells. Indirect results suggest that AhR could cooperate not only with additional transcription factors but also with membrane-associated proteins to drive such processes.

**Results:**

In this study, we have used immortalized and primary dermal fibroblasts from wild type (*AhR+/+*) and AhR-null (*AhR−/−*) mice to show that AhR modulates membrane distribution and mobilization of caveolin-1 (Cav-1) during directional cell migration. AhR co-immunoprecipitated with Cav-1 and a fraction of both proteins co-localized to detergent-resistant membrane microdomains (DRM). Consistent with a role of AhR in the process, *AhR−/−* cells had a significant reduction in Cav-1 in DRMs. Moreover, high cell density reduced AhR nuclear levels and moved Cav-1 from DRMs to the soluble membrane in *AhR+/+* but not in *AhR−/−* cells. Tyrosine-14 phosphorylation had a complex role in the mechanism since its upregulation reduced Cav-1 in DRMs in both *AhR+/+* and *AhR−/−*cells, despite the lower basal levels of Y^14^-Cav-1 in the null cells. Fluorescence recovery after photobleaching revealed that AhR knock-down blocked Cav-1 transport to the plasma membrane, a deficit possibly influencing its depleted levels in DRMs. Membrane distribution of Cav-1 in AhR-null fibroblasts correlated with higher levels of cholesterol and with disrupted membrane microdomains, whereas addition of exogenous cholesterol changed the Cav-1 distribution of *AhR+/+* cells to the null phenotype. Consistently, higher cholesterol levels enhanced caveolae-dependent endocytosis in AhR-null cells.

**Conclusions:**

These results suggest that AhR modulates Cav-1 distribution in migrating cells through the control of cholesterol-enriched membrane microdomains. Our study also supports the likely possibility of membrane-related, transcription factor independent, functions of AhR.

**Electronic supplementary material:**

The online version of this article (doi:10.1186/s12964-014-0057-7) contains supplementary material, which is available to authorized users.

## Background

The aryl hydrocarbon Receptor (AhR)/Dioxin receptor is a basic-helix-loop-helix (bHLH) transcription factor well known for its relevant role in the cellular response to carcinogens such as TCDD and benzo[*a*]pyrene [1,2]. Many studies over the last few years have also established that AhR expression is required for normal cell functioning and for the homeostasis of the hepatic, immune, cardiovascular and reproductive systems [3,4].

Particularly interesting is the implication of AhR in the control of cell adhesion and migration under normal (e.g. xenobiotic-free) conditions. Previous work has shown that the AhR-target gene *Cyp1a1* is induced after suspension of human keratinocytes, mouse Hepa1 cells and 10T1/2 fibroblasts [5,6], suggesting that AhR is activated following the disruption of cell-cell and cell-substratum interactions. In agreement, AhR knock-out altered positioning and axon migration of neuronal cells in the invertebrate *C. elegans* [7] and reduced migration of murine fibroblasts [8-10] and endothelial cells [11]. Such migration-related functions of AhR can be induced by TCDD in human hepatoma HepG2 [12] and human breast tumor MCF-7 [13] cells or by receptor knock-out in primary keratinocytes [14]. Taken together, these studies emphasize that AhR is likely a novel molecular intermediate in the signaling pathways controlling cell adhesion and migration.

Under xenobiotic-free conditions, murine fibroblasts lacking AhR (*AhR−/−*) develop an excess of F-actin stress fibers and a significant increase in the number and size of focal adhesions that compromise their migratory and invasive potentials. These phenotypes are at least partially due to the inefficient activation of focal adhesion kinase and to an altered balance between Rac1 and RhoA activities, presumably consequence of the reduced expression of the AhR target gene Vav3 [10,15]. More recently, we have described that AhR also controls cell adhesion and migration of mesenchymal fibroblasts through the extracellular matrix protein fibronectin and the membrane adaptor Cbp/Pag1 (Csk-binding protein) [16]. This latter mechanism converges to the regulation of β1-integrin and c-Src activities that ultimately controls Cav-1 expression and phosphorylation [16]. Based on this work, we proposed the existence of a pathway connecting AhR with Cav-1 in the control of cell adhesion and migration.

Cav-1 is one of the main components of the 60–80 nm plasma membrane caveolae structures [17-19] involved in the regulation of multiple cellular functions [20] and in the progression of serious diseases such as cancer [21,22]. Importantly, Cav-1 is relevant in cell migration as mouse embryonic fibroblasts (MEFs) lacking this protein have defects in cell polarity, altered directional migration and increased number of focal adhesions [23]. Furthermore, Cav-1 has a polarized distribution in migrating cells, being mostly localized to the rear part of the cell where it contributes to focal adhesion recycling and lamellipodia inhibition [24,25]. By contrast, Y^14^-phosphorylated Cav-1 moves forward to the front edge of migration where it recruits Csk and helps the generation of new adhesions [26]. Additional important functions of Cav-1 include endocytosis, intracellular cholesterol transport and mechanosensing [27].

Only very few toxicological studies have related AhR to Cav-1. In this regard, it has been shown that polychlorinated biphenyls such as PCB77 induced the AhR target gene *Cyp1a1* and *Cav-1* in endothelial cells, and that such effect seems to involve an association between AhR and Cav-1 [28,29]. These work, together with our findings suggesting that AhR modulates Cav-1 Y^14^ phosphorylation through c-Src kinase in murine fibroblasts [16], prompted us to investigate whether AhR modulates Cav-1 activities in migrating mesenchymal cells.

We report here that, indeed, AhR expression modulates the localization of Cav-1 at the cell membrane as well as its distribution between microdomains and soluble membrane in directionally migrating fibroblasts. Such effects probably depend on cholesterol levels and on the interaction between AhR and Cav-1. We propose that Cav-1 requires the AhR-dependent control of cholesterol to maintain its proper membrane distribution during cell migration.

## Results

### Caveolin-1 distribution in mouse fibroblasts is AhR dependent.

**Figure 1:**
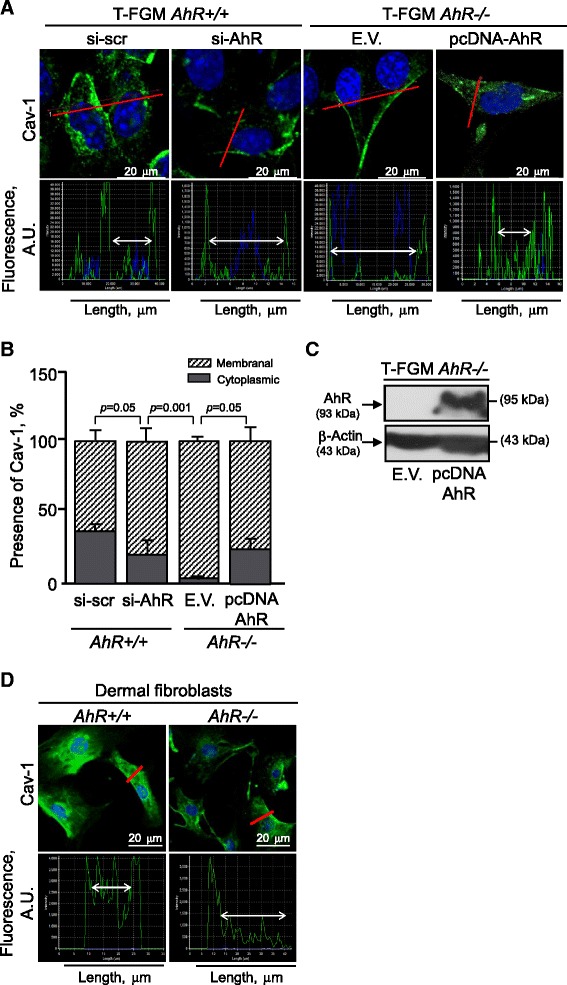
**Cav-1 has a differential location in fibroblasts lacking AhR. (A)** T-FGM *AhR+/+*fibroblasts transfected with a siRNA against AhR (si-AhR) or with an scrambled sequence, and T-FGM *AhR−/−* fibroblasts transfected with a pcDNA-AhR or with an empty expression vector (E.V.) were analyzed for Cav-1 expression by immunofluorescence using an Alexa 488-conjugated secondary antibody. **(B)** Quantification of the cellular distribution of Cav-1 in T-FGM cells. Cav-1 was considered membranal (dashed bars) when present within a 2 μm distance from the cell border or cytosolic (gray bars) when expressed from 2 μm up to the cell nucleus. Measurements were taken by triplicate in two cultures of each genotype. **(C)** The ability of the pcDNA AhR expression construct to rescue receptor expression in *AhR−/−* fibroblasts was determined by immunoblotting. **(D)** Primary dermal fibroblasts obtained from the skin of *AhR+/+* and *AhR−/−* newborn mice were also analyzed as above. Regions of interest (ROI) were selected (red line in upper panels) and the levels of fluorescence analyzed by using the “ROI profiles” tool of the Fluoview software. The intensity per pixel was plotted for Cav-1 (green). Cav-1 was detected using a Fluoview F1000 confocal microscope. The nuclear signal is represented in dark blue (DAPI staining). The experiments were done at least three times in independent T-FGM and dermal fibroblasts cultures. Data are shown as mean ± s.d.

**Figure 2:**
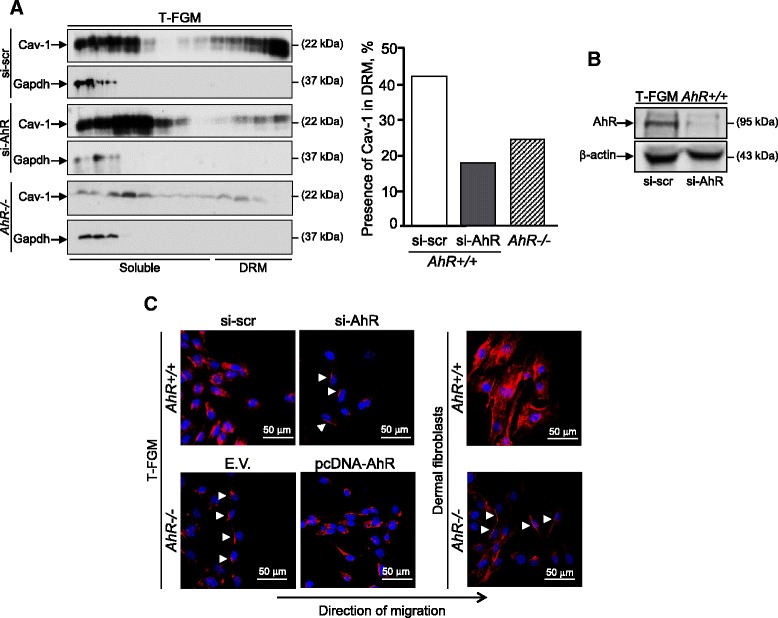
**Dioxin receptor expression modulates Cav-1 distribution in DRMs and at the rear of migrating fibroblasts. (A)** Extracts from T-FGM *AhR+/+* cells transfected with a scramble (si-scr) or with a specific siRNA for AhR (si-AhR) and extacts from T-FGM *AhR−/−* cells were analyzed by sucrose density gradient centrifugation. The presence of Cav-1 in each fraction of the gradient was analyzed by immunoblotting. Gapdh was used as a cytoplasmic control protein. Detergent resistant membrane microdomains (DRM) correspond to the lower density (upper) fractions of the gradient. The quantification of the percentage of Cav-1 located in DRM fractions with respect to the total amount of Cav-1 in a representative experiment is shown. Data were normalized to the T-FGM *AhR+/+* si-scr control condition. **(B)** RNA interference against AhR efficiently down-modulated protein expression in T-FGM *AhR+/+* fibroblasts as compared with non-specific scramble sequences (si-scr). **(C)** Basal *AhR+/+* and *AhR−/−* T-FGM fibroblasts, *AhR+/+* fibroblasts transfected with a siRNA for AhR and *AhR−/−* cells transfected with a pcDNA-AhR expression construct were grown to confluence and analyzed for directional migration using wound-healing assays. The same experiment was performed using dermal fibroblasts isolated from *AhR+/+*and *AhR−/−* newborn mice (right). Cav-1 was detected by immunofluorescence in a Fluoview F1000 confocal microscope. Cell nuclei were stained with DAPI. Arrowheads indicate rear distribution of Cav-1 in *AhR−/−* cells. The experiments were done at least three times in independent T-FGM and dermal fibroblasts cultures.

We have previously found that fibroblasts lacking AhR expression had impaired directional migration and low levels of Cav-1 Y^14^ phosphorylation, likely because their reduced c-Src activity [16]. Since Cav-1 has a relevant role in cell polarization and in directional migration [30], we decided to first determine by immunofluorescence the cellular distribution of Cav-1 under basal conditions and during directional migration. For these experiments, Cav-1 was considered to have membrane location when present within 2 μm from the cell border and cytosolic location when situated from 2 μm up to the cell nucleus. While T-FGM *AhR+/+* fibroblasts had Cav-1 scattered along the cellular periphery and the intracellular space (Figure 1A,B), T-FGM *AhR−/−*fibroblasts located Cav-1 mostly to the cell periphery, with very low intracellular levels (Figure 1A,B). Such Cav-1 distribution was AhR dependent since it could be mimicked by AhR knock-down (si-AhR) (Figure 2A) in T-FGM *AhR+/+* cells (Figure 1A,B), and because rescue of AhR expression in T-FGM *AhR−/−* fibroblasts (Figure 1C) redistributed Cav-1 to a pattern resembling that of wild type cells (Figure 1A,B). The effects of AhR on Cav-1 distribution appeared common for fibroblastic cells since it could be also observed in primary dermal fibroblasts from *AhR−/−* mice (Figure 1D). The staining of Cav-1 was specific as shown by immunofluorescences performed in the absence of specific antibody (Additional file 1: Figure S1).

Cav-1 switches between detergent resistant membrane microdomains (DRM) and the soluble membrane in migrating cells [30]. We next used sucrose density gradients to investigate whether AhR could affect the presence of Cav-1 in DRMs (Figure 2). T-FGM fibroblasts lacking AhR expression had a reduced amount of Cav-1 in membrane microdomains as compared to wild type T-FGM fibroblasts (Figure 2A, lower panels). AhR had a causal role in the membrane distribution of Cav-1 since its knock-down in *AhR+/+* T-FGM cells (Figure 2B) reduced the amount on Cav-1 in DRM fractions (Figure 2A, upper panels). Scramble si-AhR did not significantly reduce AhR levels nor had a relevant effect on Cav-1 distribution (Figure 2A,B). Cav-1 is coordinately distributed between the front and the rear in directionally migrating cells [23,24]. At the rear forms part of caveolae whereas at the front contributes to the synthesis of new focal adhesions, probably by a process requiring its phosphorylation at the Y^14^ residue [26]. We then used wound-healing assays to analyze the location of Cav-1 in directionally migrating *AhR+/+*and *AhR−/−* fibroblasts. Cav-1 mostly located at the rear in T-FGM *AhR−/−* fibroblasts whereas it was predominantly distributed at the front in T-FGM *AhR+/+* cells (Figure 2C). Such effect required AhR since its down-modulation in *AhR+/+* cells shifted Cav-1 to the rear membrane whereas its re-expression in *AhR−/−*fibroblasts rescued a wild-type like Cav-1 phenotype (Figure 2C). In addition, migrating dermal fibroblasts from *AhR−/−* mice also localized Cav-1 at the rear part of the cell (Figure 2C), further supporting the implication of AhR in the process.

### AhR is present at membrane microdomains and associates with Cav-1

**Figure 3:**
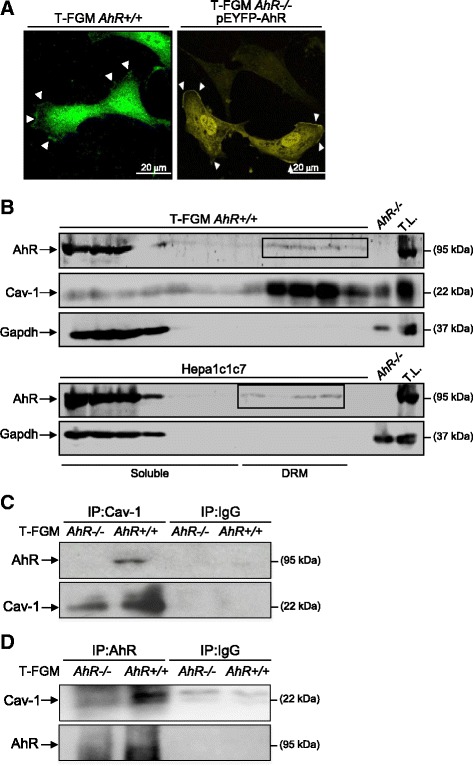
**The dioxin receptor is present in membrane microdomains and associates to Cav-1. (A)** Basal AhR distribution was determined in T-FGM *AhR+/+* cells by confocal immunofluorescence using an anti-AhR antibody bound to an Alexa 488 secondary antibody (left). T-FGM *AhR−/−* fibroblasts were transfected with a pEYFP-AhR expression vector and protein distribution analyzed by confocal microscopy using a FluoView 1000 confocal microscope. Arroheads mark AhR location (right). **(B)** Extracts from T-FGM *AhR+/+* fibroblasts and mouse hepatoma Hepa1 cells were obtained and analyzed for AhR and Cav-1 distribution by sucrose density gradients. Fractions were collected and analyzed for the presence of AhR and Cav-1 by immunoblotting. Gapdh was used as cytoplasmic control. DRM fractions containing AhR are squared. Total lysates (T.L.) from T-FGM *AhR+/+* and *AhR−/−* fibroblasts were used as positive and negative controls, respectively*.* **(C,D)** T-FGM *AhR+/+* and *AhR−/−* cells were lysed and 1 mg of total protein immunoprecipitated with anti-Cav-1 **(C)** or anti-AhR **(D)** antibodies. Immunoprecipitates were analyzed for the presence of AhR and Cav-1 by immunoblotting. Immunoprecipitation reactions were also done in T-FGM *AhR−/−* fibroblasts as negative controls. The experiments were repeated in the absence of specific antibodies (IP:IgG) to confirm specificity. Assays were done in duplicate in three different cell cultures.

**Figure 4:**
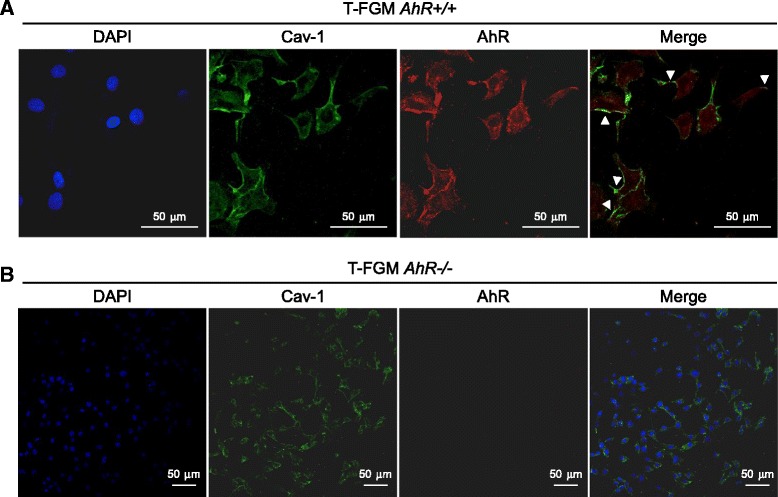
**AhR and Cav-1 co-localize at the cell periphery in fibroblast cells. (A)** T-FGM-*AhR+/+* cells were grown on glass coverslips and analyzed for the co-localization of AhR and Cav-1 by immunofluorescence using Fluoview F1000 confocal microscopy. Cells were incubated with anti-Cav1 or anti-AhR primary antibodies and then with secondary antibodies coupled to Alexa 488 or Alexa 647, respectively. Signals obtained for each individual protein have been merged on the right panel. **(B)** The same immunofluorescences were done in parallel in T-FGM *AhR−/−*fibroblasts. Note the absence of receptor expression in *AhR−/−* cells. Arrowheads in panel A indicate areas of the cell membrane with apparent AhR and Cav-1 co-localization. DAPI staining was used to label cell nuclei. The experiments were done in triplicate in two cultures of each genotype and different areas of the cultures were analyzed.

The existence of a pool of AhR associated to the plasma membrane was first suggested by the presence of the endogenous protein at the leading edge of migrating T-FGM *AhR+/+* fibroblasts and by the location of the ectopic EYFP-AhR protein in lamellipodia of the plasma membrane of T-FGM *AhR−/−* fibroblasts (Figure 3A). Since AhR expression seemed to influence Cav-1 distribution (see Figure 2), we decided to analyze if both proteins could be part of a common complex in fibroblast cells. Sucrose density gradients showed that an amount of AhR protein was found in Cav-1-containing DRM fractions of T-FGM *AhR+/+* fibroblasts (Figure 3B, upper) and, importantly, of phenotypically unrelated mouse Hepa1 hepatoma cells (Figure 3B, lower). The possible association between both proteins gained additional support by co-immunoprecipitation assays for Cav-1 (Figure 3C) and AhR (Figure 3D). The results obtained revealed that Cav-1 and AhR could immunoprecipitate each other in wild type fibroblasts, thus agreeing with our hypothesis. Consistently, AhR and Cav-1 association did not take place in *AhR−/−* fibroblasts nor it was observed in the absence of specific antibodies (Figure 3C,D). In addition, these data are coincident with a previous study reporting that AhR co-immunoprecipitates with Cav-1 in endothelial cells [28]. Nevertheless, to provide additional experimental strength to the association of AhR and Cav-1, we performed confocal microscopy co-immunofluorescence for both proteins in T-FGM *AhR+/+* fibroblasts (Figure 4A). Cav-1 had an expression pattern with a significant protein distribution at the cell periphery. AhR was mainly expressed in the cytosolic and nuclear compartments although it was also readily detectable at the cell periphery. Overlapping of both expression patterns revealed areas of the cell membrane where AhR and Cav-1 co-localized (Figure 4A, arrowheads), hence confirming their potential association into a common molecular complex. T-FGM *AhR−/−* fibroblasts used as negative controls did not show any AhR expression or Cav-1 co-localization (Figure 4B). The existence of a fraction of AhR functionally associated to the plasma membrane was also supported by its co-localization with the cell adhesion molecule talin (results not shown), which, in fact, has a role in the AhR-dependent phenotype of cell adhesion [15,16].

### AhR expression and cell density modulate Cav-1 distribution

**Figure 5:**
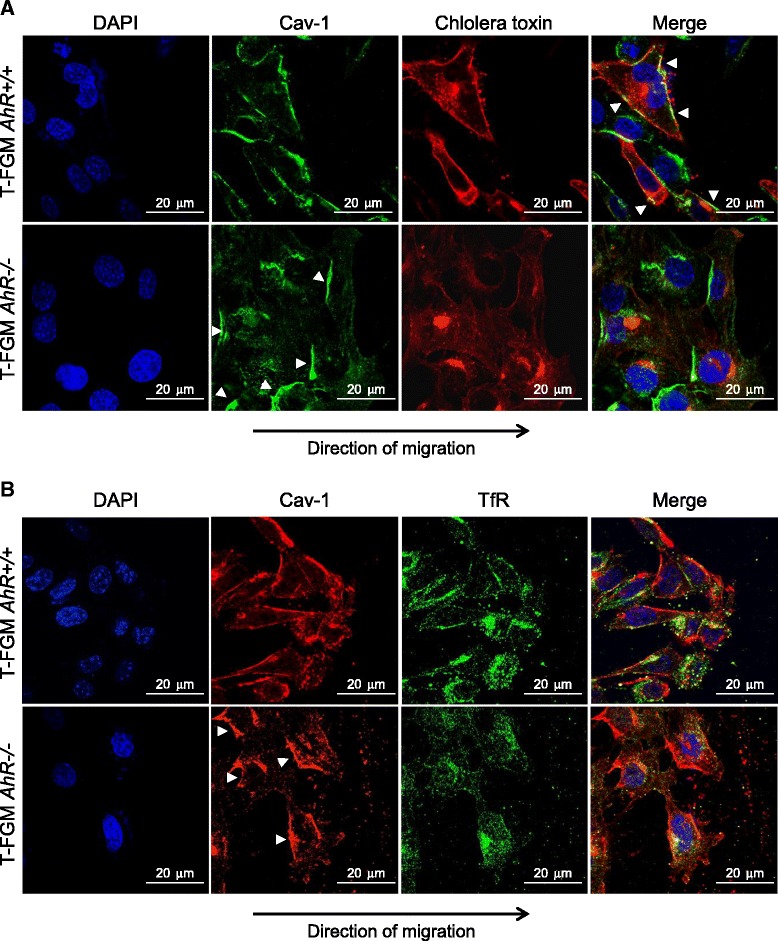
**AhR contributes in determining Cav-1 localization to DRM domains in directionally migrating fibroblasts. (A)** T-FGM *AhR+/+* and *AhR−/−* fibroblasts were grown to confluence on glass coverslips. Wounds were done to induce directional migration (indicated by an arrow). The expression patterns of Cav-1 and cholera toxin β (DRM marker) were analyzed by immunofluorescence using a Fluoview F1000 confocal microscope. Cav-1 and cholera toxin β were detected using secondary antibodies conjugated with Alexa 488 and Alexa 633, respectively. A merge of both expression profiles is shown on the right. **(B)** The same experiments were performed to determine the co-localization of Cav-1 with the soluble membrane marker TfR. Note that Cav-1 was detected using a secondary antibody labeled with Alexa 633 whereas TfR was bound to an Alexa 488-labelled secondary antibody. DAPI staining was used to label cell nuclei. Arrowheads indicate co-localization of Cav-1 with cholera toxin β in T-FGM *AhR+/+* cells or Cav-1 in T-FGM *AhR−/−* cells. The experiment was done in triplicate in two T-FGM cultures of each genotype.

Despite the apparent co-localization of AhR and Cav-1 in membrane fractions of fibroblasts, sucrose density gradients could not discriminate whether AhR influences Cav-1 distribution between DRMs and the soluble membrane. To address this question, we used immunofluorescence to analyze in directionally migrating *AhR+/+* and *AhR−/− *fibroblasts the co-localization of Cav-1 with cholera toxin β as a DRM marker and with the transferrin receptor (TfR) as a representative soluble membrane protein (Figure 5). Cav-1 co-localized with cholera toxin β in the front and rear areas of the plasma membrane of T-FGM *AhR+/+* cells. Notably, such co-localization was not observed in T-FGM *AhR−/−* fibroblasts, which exhibited their typical expression pattern of Cav-1 at the rear part of the cell and a marked de-localization of cholera toxin β (Figure 5A). On the contrary, Cav-1 did not co-localize with TfR in T-FGM fibroblasts of either genotype, showing the transferrin receptor a classical punctate pattern along the cell (Figure 5B). These results suggest that, indeed, AhR has a role in determining Cav-1 distribution to DRMs in fibroblast cells.

**Figure 6:**
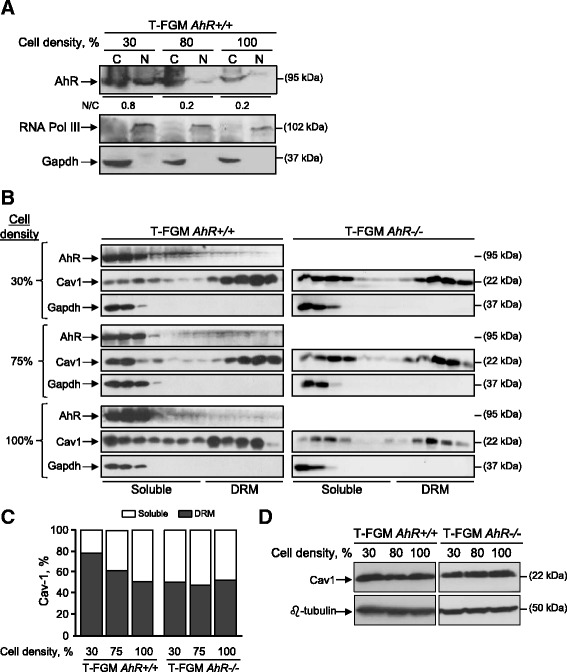
**Cell density modulates Cav-1 distribution in an AhR-dependent manner. (A)** T-FGM *AhR+/+*cells were cultured at different cell densities from low (30%) to high (100%) confluence and the presence of the AhR protein analyzed by immunoblotting in nuclear “N” and cytosolic “C” extracts. The catalytic subunit of the RNA polymerase III and Gapdh were used as markers for the nuclear and cytosolic compartments, respectively. The ratio of nuclear:cytosolic AhR is indicated below the blot. **(B)** T-FGM *AhR+/+* and *AhR−/−* fibroblasts cultured at different confluences were used to obtain protein extracts that were analyzed for Cav-1 and AhR distribution using sucrose density gradients. Gapdh was used as a marker for the soluble fractions. The presence of each protein was determined by immunoblotting using specific antibodies. **(C)** The content of Cav-1 in DRMs and soluble fractions of T-FGM *AhR+/+* and *AhR−/−* fibroblasts was quantified and plotted for each cell density. At least 4 cultures were used for each experimental condition and cell genotype. A representative experiment and its quantification are shown. **(D)** Total cell extracts obtained from T-FGM *AhR+/+* and *AhR−/−* fibroblasts grown at 30% to 100% confluence were analyzed for AhR expression by immunoblotting. β-tubulin was used to confirm equal loading and protein integrity. Determinations were done in duplicate in two cultures of each genotype.

The amount of AhR that is present in the nuclear and cytoplasmic compartments of the cell is modulated by cell density in keratinocytes [31] and hamster fibroblasts [5]. In those cell types, AhR is predominantly nuclear at sparse cell densities and cytoplasmic at confluence. Since AhR modulates Cav-1 distribution in T-FGM fibroblasts, we next investigated if the intracellular localization of AhR affected the fraction of Cav-1 associated to DRMs. T-FGM *AhR+/+* fibroblasts grown at low cell density (e.g. 30% confluence) had AhR distributed between the nucleus and the cytosol at a 0.8:1 ratio; as cell density increased to full confluence, AhR levels were significantly reduced in the nuclear compartment (Figure 6A). Sucrose density gradients of T-FGM *AhR+/+* cell extracts revealed that Cav-1 moved from DRM to soluble membrane fractions at elevated cell densities (Figure 6B left, 6C), an effect that was coincident with an increased ratio in the cytosolic *vs* nuclear AhR. In agreement with these results, *AhR−/−* fibroblasts growing at high cell density did not significantly change Cav-1 distribution between DRM fractions and the soluble membrane (Figure 6B right, 6C). The effects of cell density on Cav-1 distribution were not due to differences in Cav-1 protein levels as shown by immunoblotting analyses of T-FGM *AhR+/+* and *AhR−/−* cells cultured from 30% to 100% confluence (Figure 6D).

### Phosphorylation at Y^14^ modifies Cav-1 distribution but it is AhR-independent

**Figure 7:**
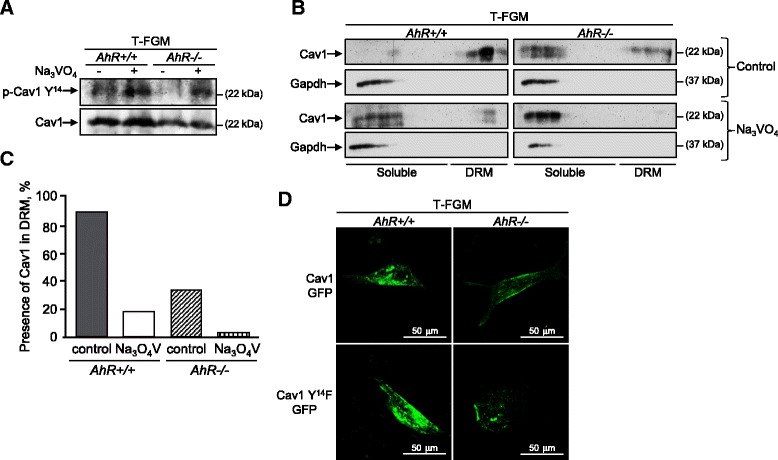
**Y** ^**14**^ **phosphorylation does not have a definitive role in Cav-1 distribution upon AhR expression. (A)** T-FGM *AhR+/+* and *AhR−/−*fibroblasts were treated for 1 h with the phosphatase inhibitor Na_3_VO_4_ or with solvent. Total protein extracts were analyzed by immunoblotting with specific antibodies against total and phosphorylated Cav-1-Y^14^ protein. **(B)** T-FGM cells of both genotypes were treated with Na_3_VO_4_ or with solvent and cellular extracts analyzed for Cav-1 distribution by sucrose density gradients. Immunoblotting was used to detect Cav-1 and the cytosolic marker Gapdh. **(C)** Cav-1 was quantified in DRMs and in soluble membrane fractions and the results represented with respect to the total amount of Cav-1 in the gradient. A representative experiment is shown. **(D)** T-FGM *AhR+/+* and *AhR−/−* fibroblasts were grown on glass coverslips and transfected with a wild type Cav-1-GFP or a Cav-1-Y^14^F-GFP non-phosphorylable mutant. Cav-1 distribution was analyzed by live cell microscopy using a CellR fluorescence equipment. The experiments were done in duplicate in two cultures of each cell genotype.

Phosphorylation at Y^14^ has been proposed to modulate several functions of Cav-1 including caveolae internalization and the formation of new focal adhesions [32,33]. Since T-FGM *AhR−/−* cells have a significant reduction in Cav-1 Y^14^ phosphorylation [16], we have analyzed whether this posttranslational modification could be involved in modulating Cav-1 distribution in AhR expressing cells. Treatment with the protein phosphatase inhibitor Na_3_VO_4_ significantly increased the levels of Cav-1 Y^14^ phosphorylation in both T-FGM *AhR+/+* and *AhR−/−* fibroblasts without significantly affecting total Cav-1 protein expression (Figure 7A). Sucrose density gradients revealed that Na_3_VO_4_ reduced the content of Cav-1 in DRM fractions of both T-FGM *AhR+/+* and *AhR−/−* fibroblasts (Figure 7B,C), indicating that an increase in phosphorylation moves Cav-1 to the soluble membrane. To try to further support this result, we transiently transfected T-FGM *AhR+/+* and *AhR−/−* cells with wild type Cav-1-GFP and with a Cav-1-Y^14^F-GFP non-phosphorylable mutant. Fluorescent confocal microscopy revealed that Cav-1-GFP wild type and the Cav-1-Y^14^F-GFP mutant had similar cellular distribution in either *AhR+/+*or *AhR−/−* T-FGM fibroblasts (Figure 7D), arguing that Y^14^ phosphorylation may not be essential in Cav-1 distribution upon AhR expression.

### Cav-1 has reduced mobility in AhR-null fibroblasts

**Figure 8:**
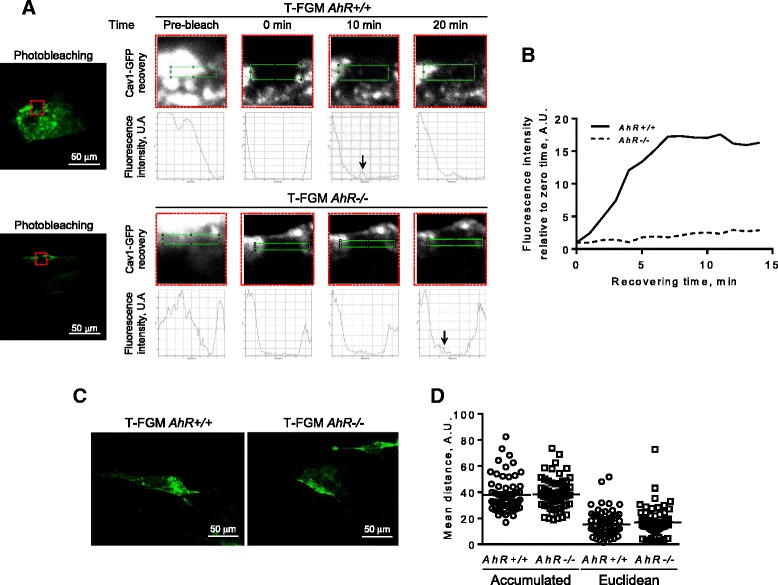
**AhR is required to recruit Cav-1-containing vesicles to the membrane. (A)** T-FGM *AhR+/+* and *AhR−/−* fibroblasts were transfected with a Cav-1-GFP fusion protein and its recruitment to the membrane analyzed by fluorescence recovery after photobleaching (FRAP) using live cell microscopy. Images were taken before (pre-bleach) and up to 20 min after bleaching. The bleached area is indicated with a red square on the left panels. The intensities of Cav-1-GFP signals were analyzed in selected areas (green rectangles) and the profiles obtained are shown in the lower panels. Vertical arrows mark fluorescence recovery. **(B)** Fluorescence recovery for cells of each genotype was quantified with respect to zero time. **(C)** T-FGM *AhR+/+* and *AhR−/−* cells were transfected with a Cav-1-GFP expression vector and the distance covered by the vesicles analyzed by live cell microscopy under controlled conditions. Vesicle movement was determined by taking one image on the Z axis for each millimeter during 20 min. Representative images correspond to the projection along the Z axis of pictures taken at one fixed time. **(D)** The accumulated and the Euclidean distances covered by Cav-1-GFP vesicles were measured and plotted for T-FGM *AhR+/+* and *AhR−/−* cells. At least 15 vesicles from at least 3 different cells were analyzed. The assays were done in triplicate cultures of each cell line.

Membrane microdomains have a relevant role in caveolae stabilization [34] and Cav-1 is one of the main intracellular transporters of the DRM component cholesterol [35]. Based on the previous results, we decided to analyze if the altered distribution of Cav-1 in AhR lacking cells could be reflecting defects in its mobilization and recruitment to the plasma membrane. Fluorescence recovery after photobleaching (FRAP) was done in T-FGM *AhR+/+* and *AhR−/−* fibroblasts transfected with a Cav-1-GFP fusion protein (Figure 8A,B). Transport of Cav-1-GFP to the cell membrane reached its maximum value 8–10 min after bleaching in T-FGM *AhR+/+* cells whereas only a marginal recovery could be observed in *AhR−/−* fibroblasts after 20 min of bleaching. However, such impaired recovery was not due to a reduced ability of Cav-1-loaded vesicles to migrate. Quantification by live cell microscopy of the movement of Cav-1-GFP loaded vesicles (Figure 8C) revealed that the accumulated and the Euclidean distances covered by Cav-1 vesicles were very similar in T-FGM *AhR+/+* and *AhR−/−* fibroblasts (Figure 8D). Therefore, AhR appears to be needed for an efficient Cav-1 transport to the membrane although it does not seem to affect the net distance traveled by the vesicles.

### Cholesterol is increased in the absence of AhR and it regulates Cav-1 localization

**Figure 9:**
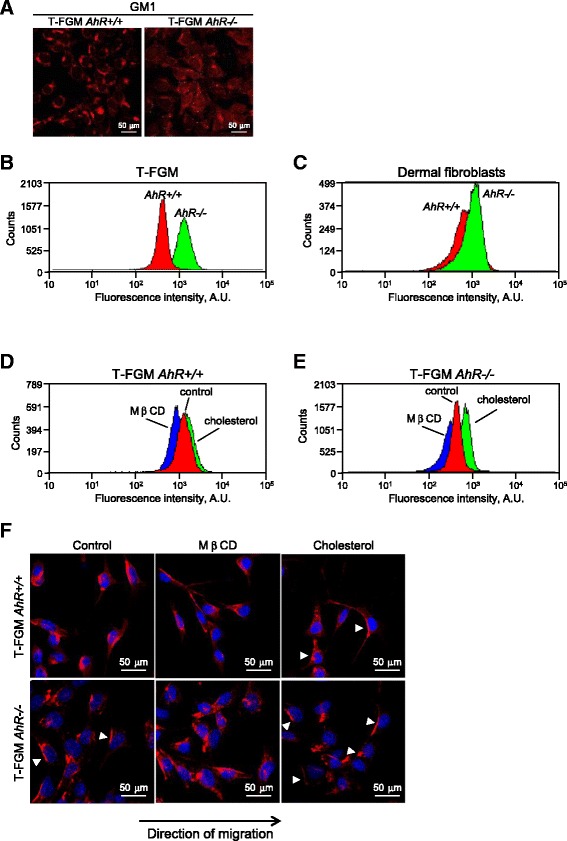
**AhR modulates basal cholesterol levels in fibroblasts. (A)** T-FGM *AhR+/+* and *AhR−/−* cultures were stained with the cholesterol dye M1 ganglioside (GM1) and membrane microdomains analyzed by fluorescence confocal microscopy. **(B,C)** T-FGM cells **(B)** and primary dermal fibroblasts **(C)** of both genotypes were grown on glass coverslips, fixed and stained with the cholesterol-binding antibiotic filipin III in order to detect endogenous free cholesterol. Stained cells were analyzed by flow cytometry and the fluorescence intensity profiles represented. **(D,E)** T-FGM *AhR+/+* and *AhR−/−* cells were incubated for 16 h with 10 mM MβCD, 100 mM cholesterol plus 2.5 mM MβCD (cholesterol) or solvent and analyzed for cholesterol content by flow cytometry as indicated above. Fluorescence profiles were compared graphically. **(F)** T-FGM cells of both genotypes were grown to confluence and treated with MβCD or cholesterol as indicated above. Wound healing was used to induce directional migration. Cav-1 distribution was analyzed by fluorescence confocal microscopy in a Fluoview F1000 equipment. DAPI staining was used to label cell nuclei. Arrowheads mark Cav-1 location. The experiments were done in duplicate in two cultures of each genotype.

Cholesterol is considered a regulator of Cav-1 transport between the Golgi cisternae and the plasma membrane [36] and a previous study has shown that AhR activation by TCDD inhibits the expression of cholesterol biosynthetic enzymes [37]. We thus investigated whether AhR modulates basal cholesterol levels and if cholesterol is involved in Cav-1 distribution in migrating cells. DRMs are in a large part composed of gangliosides and cholesterol [38]. While T-FGM *AhR+/+*cells had a well-defined membrane distribution of the GM1 ganglioside as determined by cholera toxin β staining, T-FGM *AhR−/−* fibroblasts showed a marked delocalization of GM1 (Figure 9A). Measurement of cholesterol levels by flow cytometry using the cholesterol-binding antibiotic filipin [39] revealed that T-FGM *AhR−/−* fibroblasts had a large increase in cholesterol content with respect to *AhR+/+* cells (Figure 9B). The same effect was also found in primary dermal fibroblasts from *AhR−/−* mice, further supporting that AhR is involved in cholesterol maintenance (Figure 9C). To determine if cholesterol plays a role in Cav-1 distribution, T-FGM cultures under directional migration were treated with methyl-β-cyclodextrin (MβCD) to disrupt cholesterol-enriched membrane microdomains or with exogenous cholesterol. Flow cytometry confirmed that MβCD effectively reduced the basal cholesterol content whereas the addition of exogenous cholesterol increased its levels in T-FGM *AhR+/+* and *AhR−/−* fibroblasts (Figure 9D,E). The results showed that T-FGM *AhR+/+* fibroblasts treated with exogenous cholesterol re-localized Cav-1 to the rear part of the cell whereas *AhR−/−* fibroblasts treated with MβCD de-localized Cav-1 from the rear to the front edge of the cell (Figure 9F). Thus, an increase in cholesterol could switch the pattern of Cav-1 distribution of *AhR+/+* cells to the AhR-null-like phenotype. Conversely, disruption of membrane microdomains by MβCD induced a wild type-like distribution of Cav-1 in *AhR−/−* cells. Sucrose density gradients could not be performed under these experimental conditions since MβCD provoked a drastic reduction in the amount of Cav-1 in DRM fractions, perhaps because of an almost complete depletion of cholesterol, as reported [40].

### Caveolae endocytosis is enhanced in fibroblasts lacking AhR

**Figure 10:**
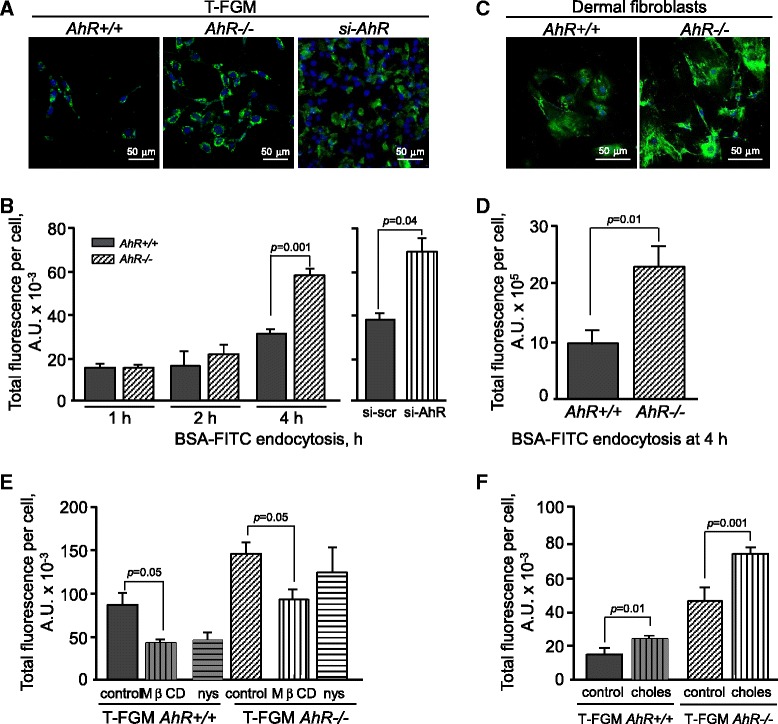
**Lack of AhR increases caveolae-dependent endocytosis. (A)** Basal T-FGM *AhR+/+* and *AhR−/−* fibroblasts and T-FGM *AhR+/+* cells transfected with a si-AhR were grown on glass coverslips for 24 h. Cells were then incubated with serum-free medium for 2 h and with BSA-FITC for 1, 2 or 4 h. Cultures were observed in a Fluoview F1000 fluorescent confocal microscope. Cell nuclei were stained with Hoechst 33342. **(B)** Total fluorescence per cell was digitally calculated with the ImageJ software and represented for each treatment. **(C)** The same assay was performed in dermal fibroblasts from *AhR+/+* and *AhR−/−* newborn mice measuring endocytosis after 4 h of treatment. **(D)** Total fluorescence per cell was digitally calculated with the ImageJ software. **(E)** T-FGM cells of both genotypes were treated with 10 mM MβCD, 15 mg/ml nystatin or solvent (control) for 1 h prior to the incubation with BSA-FITC for 4 h. Endocytosis was quantified and analyzed as indicated above. **(F)** T-FGM *AhR+/+* and *AhR−/−* cells were pre-treated with 100 mM cholesterol plus 2.5 mM MβCD (choles) or solvent (control) for 16 h prior to the incubation with BSA-FITC for 4 h. Endocytosis was quantified and analyzed as indicated above. Data are shown as mean ± s.d. from experiments performed in duplicate in three independent cultures.

The fact that AhR deficiency alters Cav-1 distribution at DRMs, and since caveolae disruptors such as MβCD are cholesterol sequesters [41], lead us to investigate the existence of alterations in endocytosis, a process in which Cav-1 has a relevant role [34,42]. We used the classical caveolae-dependent cargo protein BSA-FITC [43,44] to perform endocytosis assays in T-FGM *AhR+/+*and *AhR−/−* cells. A significant increase in BSA-FITC endocytosis was observed at 4 h in T-FGM *AhR−/−* with respect to *AhR+/+* cells (Figure 10A,B). Notably, AhR knock-down in T-FGM *AhR+/+* cells increased BSA-FITC endocytosis to a comparable extent to that found in *AhR−/−* cells (Figure 10A,B). A similar effect could be also observed in primary dermal fibroblasts from AhR-null mice (Figure 10C,D), indicating that AhR has a role in caveolae-driven endocytosis. The addition of the caveolae specific disruptors MβCD and nystatin significantly reduced BSA-FITC endocytosis in cells of both genotypes, thus confirming the implication of Cav-1 in the process (Figure 10E). Consistently, addition of exogenous cholesterol, a specific inducer of caveolae-dependent endocytosis [45], increased endocytosis rates in both T-FGM *AhR+/+* and *AhR−/−* cells (Figure 10F), again supporting a role for cholesterol in caveolae endocytosis and in the distribution of Cav-1 in membrane microdomains.

## Discussion

Among the recently discovered physiological functions of AhR [3], its contribution to the control of cell adhesion and migration is attracting considerable interest. We have shown that AhR has novel functions in mesenchymal fibroblasts cells by controlling the number and size of focal adhesions and the stability of actin stress fibers [3,10,15]. More recently, we have described a mechanism integrating AhR in cell adhesion and migration through the regulation of the Cbp-Csk-Src pathway ultimately leading to β1 integrin activation [16]. Interestingly, the functional interaction between AhR and Src-dependent signaling has been also suggested by other authors as AhR activation by TCDD increases c-Src activity in human MCF-10A and HepG2 cells [12,46]. Notably, these studies open the possibility to the existence of membrane-related functions of AhR that could be independent from its activity as a transcription factor.

A major finding of this work is the identification of a fraction of AhR associated with plasma membrane microdomains and in apparent co-localization with Cav-1, a protein involved in cell migration and a relevant component of the c-Src and β1-integrin signaling pathways [23,30]. Interestingly, a recent work has shown that exogenous coplanar polychlorinated biphenyls induced the co-immunoprecipitation of AhR and Cav-1 in endothelial cells [28], suggesting that the association between both proteins could be a general regulatory mechanism defining a novel signal transduction pathway.

AhR deficiency altered Cav-1 distribution in the cell not only by inducing its accumulation at the cell periphery but also by shifting its localization between the front and the rear edges of the plasma membrane. Cav-1 has relevant roles in cell polarization and in directional migration and they are in part due to its differential localization in the cell [25,30,47]. During cell migration, a fraction of Cav-1 locates to the rear part of the cell, where it participates in focal adhesion recycling and in cell contractility. The Cav-1 that accumulates at the leading edge of migrating cells contributes to the formation of new focal adhesions and appears to be phosphorylated and unrelated to caveolae [26]. Notably, AhR deficient fibroblasts under directional migration accumulated most of their Cav-1 at the rear membrane in detriment of the leading edge, an effect that could help explain their increased adhesion and lower migration rates [15,16]. The functional association between AhR and Cav-1 in the control of cell migration gains additional support by the following observations: (i) AhR is present at the plasma membrane of migrating cells and a fraction of this protein co-localizes with Cav-1; (ii) in presence of AhR, Cav-1 is preferentially distributed to plasma membrane DRMs; (iii) AhR and Cav-1 co-immunoprecipitate into a presumable common protein complex; (iv) increasing the cytosolic *vs* nuclear ratio of AhR enriches Cav-1 content in DRMs, and (v) AhR knock-out produces the opposite phenotypes in fibroblast cells. It is therefore likely that a pool of membrane-related AhR interacts with Cav-1 to regulate cell adhesion and migration.

Previous studies reported that cell density modulates the intracellular localization of AhR in keratinocytes [31] and in 10T1/2 fibroblasts [5] so that low cell densities kept AhR in the nucleus while higher confluences induced its accumulation in the cytoplasm. Based on these studies, we hypothesized that modifying the intracellular localization of AhR through changes in cell density should affect the membrane distribution of Cav-1. In agreement, as cell density increased, AhR was predominantly cytosolic and Cav-1 shifted its distribution from DRMs to the soluble membrane. It could be considered that the reduced migration observed in confluent fibroblasts may involve Cav-1 mobilization to the soluble membrane in parallel to the accumulation of cytosolic AhR. The plausible influence of cytosolic AhR on Cav-1 distribution to the soluble membrane gains additional support from the fact that *AhR−/−* fibroblasts did not exhibit changes in Cav-1 distribution upon increasing cell density.

Phosphorylation at Y^14^ has been associated to Cav-1 localization and internalization in response to diverse stimuli including shear stress [33] and partial hepatectomy [32]. An increase in Y^14^ phosphorylation of Cav-1 reduced its DRM levels in both AhR-expressing and AhR-lacking fibroblasts, initially suggesting that phosphorylation is relevant to the mechanism. However, a non-phosphorylable Y^14^F Cav-1 mutant had a membrane distribution similar to that of the wild type protein, suggesting that phosphorylation may not be essential to the process. In addition, the fact that, despite their lower basal levels of Y^14^ Cav-1 phosphorylation, *AhR−/−* fibroblasts efficiently reduced their Cav-1 content in DRMs upon Na_3_VO_4_ treatment suggest the existence of an still not clarified intricate mechanism regulating that process. Given that the role of phosphorylation on Cav-1 function is in many aspects only partially known, further work is needed to elucidate the phenotype of AhR-null cells and to what extent Y^14^ phosphorylation is relevant in modulating Cav-1 localization in fibroblasts.

Recruitment to DRMs is essential for Cav-1 to control cell migration [34]. Real time FRAP experiments showed that *AhR−/−* cells were less capable of mobilizing Cav-1 containing vesicles to the plasma membrane. Such deficient recruitment of Cav-1 could be due to impaired cytoskeleton-dependent transport and/or to altered endocytosis. However, impairment in cytoskeleton-associated transport could be excluded because the potential of Cav-1 vesicles to travel inside the cell was not affected by AhR expression. Endocytosis is a process functionally linked to membrane microdomains and, in this regard, not only Cav-1 is needed for the endocytic recycling of membrane proteins [47], but also the membrane microdomains are required for caveolae-dependent endocytosis [18]. T-FGM and primary dermal fibroblasts lacking AhR showed increased endocytosis of the Cav-1 cargo protein BSA-FITC that was partially blocked by caveolae disruptors and potential cholesterol sequesters MβCD and nystatin [48]. Cav-1 functions are tightly related to cholesterol as Cav-1 is the main carrier of that lipid [42] and because cholesterol stabilizes Cav-1-rich caveolae [19]. Consistent with a role of AhR in regulating Cav-1 through cholesterol, we have found that *AhR−/−* fibroblasts had increased levels of endogenous cholesterol and an enhanced endocytic response to exogenous cholesterol that could account for their more efficient caveolae-dependent endocytosis. Therefore, cholesterol could be an intermediate molecule in the signaling from AhR to Cav-1. This hypothesis agrees with a previous study showing that AhR activation by TCDD inhibits cholesterol biosynthesis in hepatic cells [37]. Nevetheless, more work needs to be performed to fully understand the reduced presence of Cav-1 in DRM fractions of *AhR−/−* cells given that cholesterol is a major component of DRMs and that these cells have increased caveolae-dependent endocytosis. From a functional point of view, cholesterol was also involved in AhR-dependent Cav-1 distribution during directional migration since disruption of membrane microdomains rescued *AhR−/−* cells to a wild-type-like phenotype whereas exogenous cholesterol did the opposite and induced an AhR-null-like Cav-1 phenotype in *AhR+/+* fibroblasts. Our data are consistent with previous studies showing that a high cholesterol content reduces the mobility of membrane proteins [49] and disorganizes membrane microdomains [50].

**Figure 11:**
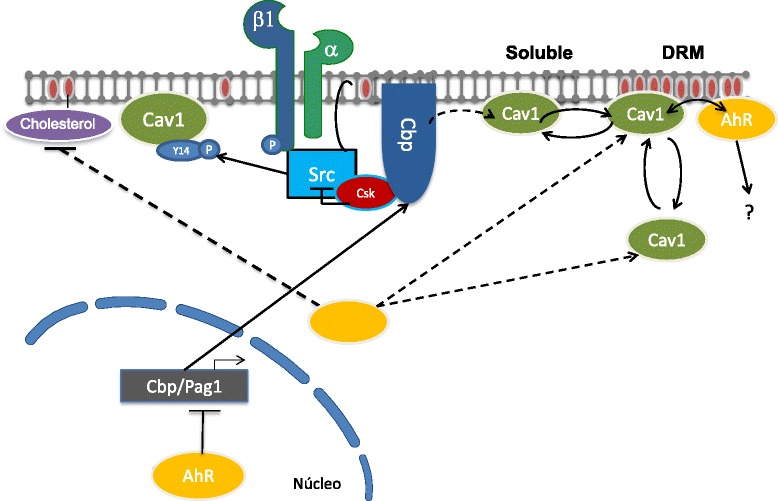
**Schematic representation of the signaling pathway proposed for the AhR-dependent control of cell migration.** The regulation of c-Src and β1-integrin through the AhR target gene *Cbp/Pag1* has been reported in our previous study [16]. Here we propose that a certain amount of cytosolic AhR locates in the vicinity of the plasma membrane during cell migration. This membrane-related AhR associates into a common protein complex with Cav1 and favors its enrichment in DRMs of the plasma membrane. It is likely that this functional relationship between AhR and Cav-1 helps establish a suitable Cav-1 distribution at the leading edge of directionally migrating fibroblasts. Cholesterol probably has a relevant role in this mechanism because it is a major lipid in defining DRM regions in the plasma membrane and because its levels are controlled by AhR. The importance of phosphorylation in determining the localization of Cav-1 is not yet clearly defined despite being a target of c-Src kinase in T-FGM fibroblasts [16].

In summary, we report here that the dioxin receptor is a novel regulator of caveolin-1 distribution and function in migrating mouse fibroblasts (Figure 11). Such effects likely involve a subpopulation of DRM-related AhR with the potential to associate to Cav-1. Cholesterol appears an effector of the Cav-1 phenotype probably by regulating caveolae-dependent endocytosis during directional migration. Thus, AhR and Cav-1 could be acting in concert to modulate cell migration during normal and pathological situations.

## Conclusions

This study reveals that AhR has a role in controlling the membrane distribution of Cav-1 during fibroblast cell migration (Figure 11). Importantly, such effect seems to take place by a mechanism involving cholesterol-enriched membrane microdomains. The functional interaction between AhR and Cav-1 likely has an important contribution to the motility of mesenchymal cells, and emphasizes the relevance of both proteins in the migration of tumor cells. Moreover, AhR and Cav-1 could have a coordinated mechanism of co-localization to adjust the rates of migration to different cellular conditions, under both physiological and pathological situations.

## Materials and methods

### Cell culture

Immortalized wild type (*AhR+/+*) and AhR-null (*AhR−/−*) T-FGM mouse fibroblasts were produced as described [10] using primary cells from *AhR+/+* and *AhR−/−* mice [51]. They were grown in DMEM/F12 medium containing 10% FBS, 2 mM L-glutamine, 50 μg/ml gentamycin and 11 mM D-glucose at 37°C in a 5% CO_2_ atmosphere. Dermal fibroblasts were obtained from the skin of 2-days-old *AhR+/+* and *AhR−/−* newborn mice following established protocols [52,53]. Briefly, the skins were floated on concentrated trypsin and the dermal layers were isolated from the epidermises. Dermises were then minced and trypsinized and the resulting cell suspension was quickly centrifuged. The cellular supernatant was removed and cultured in D-MEM medium. Mice experiments were approved by the Bioethics and Biosecurity Commission of the University of Extremadura. Mice had free access to water and rodent chow.

### Antibodies and reagents

Anti-AhR antibodies were from Biomol (Plymouth, PA, USA) or Santa Cruz Biotechnology (immunofluorescence, Santa Cruz, CA, USA); anti-β-actin was from Sigma-Aldrich (St. Louis, MO, USA) and anti-Cav-1, anti-Y^14^ p-Cav-1 and anti-Gapdh were from Becton-Dickinson (Franklin Lakes, NJ, USA). Protein A/G plus Sepharose was from Santa Cruz Biotechnology (Santa Cruz, CA, USA). Small interfering RNAs (siRNA) and scramble sequences for AhR were from Dharmacon (Lafayette, CO, USA). Cholera toxin-β to stain ganglioside GM1 was from Sigma and the anti-transferrin receptor (TfR) antibody was from Novus Biologicals (Littletown, CO, USA). The pcDNA-AhR expression vector was produced and characterized essentially as indicated [52].

### Co-immunoprecipitation and immunoblotting

Protein immunoprecipitation using T-FGM *AhR+/+* and *AhR−/−* cultures was performed essentially as described [54,55]. In brief, cells were lysed in a solution containing 50 mM Tris HCl pH 7.4, 125 mM NaCl, 2 mM DTT, 50 μM EGTA, 1 mM phenyl-methyl sulfonyl fluoride, 1% Nonidet P-40 and 4 μg/ml Complete protease inhibitor cocktail (Roche). Aliquots of 1 mg protein were incubated with 2 μg of anti-AhR antibody and protein-A/G plus Sepharose beads overnight at 4°C. Beads were then washed twice with buffer A (20 mM Tris–HCl pH 7.4, 50 mM NaCl, 1% Nonidet P-40, 10 mM EDTA, 1 mM sodium orthovanadate, 50 mM NaF, 0.5 mM PMSF and 4 μg/ml Complete protease inhibitor cocktail) and buffer B (25 mM Tris–HCl pH 7.5, 150 mM NaCl and 1 mM EDTA). Immunoprecipitated proteins were analyzed by immunoblotting as described [55].

### Transient transfection and RNA interference

T-FGM cells were transiently transfected by nucleofection using a MicroPorator MP-100 (Digital-Bio) as previously indicated [56]. The AhR-EYFP (enhanced yellow fluorescent protein) expression vector was produced by cloning the full-length murine AhR cDNA into the pEYFP vector as indicated [53]. The pEGFP (enhanced green fluorescent protein)-Cav-1 and the non-phosphorylable pEGFP-Cav-1-Y^14^F expression constructs were generously provided by Dr. Lisardo Boscá (Instituto de Investigaciones Biomédicas, Madrid, Spain). Expression vectors AhR-EYFP, pEGFP-Cav-1 and pEGFP-Cav-1-Y^14^F were used at 3 μg/ml per 10^6^ cells and experiments were performed 24–48 h after transfection. RNA interference for AhR was performed by transient transfection of specific small interfering RNAs (siRNA) or scrambled sequences (scr-RNA) at concentrations ranging from 20 nM to 100 nM.

### Discontinuous sucrose density gradients

To analyze protein distribution at detergent-resistant membrane microdomains (DRM), T-FGM *AhR+/+*and *AhR−/−* cells were collected in PBS, centrifuged and solubilized for 30 min a 4°C with gentle rotation in TNET buffer containing 50 mM Tris–HCl pH 7.5, 150 mM NaCl, 5 mM EDTA, 0.25% Triton X-100, 1 mM PMSF and 4 μg/ml Complete protease inhibitor cocktail (Roche). Cell lysates were then mixed with a 90% sucrose solution and deposited at the bottom of an ultracentrifuge tube. Samples were consecutively layered with 3.5 volumes of 35% sucrose and with 1 volume of 16% sucrose (dissolved in TNET buffer). Centrifugation was performed at 180.000 *g* for 16 h at 4°C in a Beckman-Coulter L-90K ultracentrifuge. Following centrifugation, aliquots of 200 μl were collected in such a way that DRM-enriched fractions corresponded to those located at the top of the gradient.

### Immunofluorescence and live cell microscopy

T-FGM and primary dermal fibroblasts cultures were fixed for 20 min at room temperature in 4% paraformaldehyde (Polysciences Inc) or for 10 s in ice-cold methanol. For immunofluorescence, cultures were incubated with primary antibodies for AhR or Cav-1 and then with the appropriate secondary antibody labeled with Alexas 488, 633 or 647. Cells were washed and analyzed in a Fluoview 1000 confocal microscope (Olympus). The cellular distribution of AhR-EYFP and EGFP-Cav-1 labelled proteins was also analyzed in a Fluoview 1000 confocal microscope (Olympus). To analyze Cav-1 during directional cell migration, wounds were performed with a pipette tip in confluent T-FGM cultures and cells were fixed, processed and observed 8–16 h later. For live cell microscopy, T-FGM fibroblasts growing on glass coverslips were transfected with EGFP-Cav-1 or incubated with CTB-alexa633 for DRM staining. Next, Hoechst 33342 was added to label cell nuclei and cultures were followed under controlled temperature and humidity in an Olympus CellR fluorescence microscope. In some experiments, DAPI was used to stain cell nuclei. Fluorescence distribution analysis was performed using the FV1000 software (Olympus). Objectives used were 60x oil immersion (NA 1.35) and 40x (NA 1.05). Excitation wavelengths were: 405 nm (DAPI), 488 nm (Alexa 488 and EYFP), 647 (Alexa 647) and 633 (Alexa 633). Cells stained only with secondary antibody were used as negative controls.

### Free cholesterol measurements

T-FGM and primary dermal fibroblasts were fixed with 2% paraformaldehyde for 10 min, washed with PBS and incubated for 30 min at room temperature with 5 μg/ml of the cholesterol-binding filipin III (Sigma). After propidium iodine staining to eliminate cellular debris, cultures were analyzed by flow cytometry in a Mo-Flo XDP (Beckman-Coulter) equipped with a 355 nm UV laser to excite filipin III and a 488 nm solid-state laser to excite propidium iodide (PI). A number of 10^4^ cells were analyzed per experimental condition.

### Modulation of basal cholesterol levels

The effects of cholesterol in endocytosis were analyzed by depleting or increasing its basal concentration. Basal cholesterol levels were reduced by treating T-FGM fibroblasts with 10 mM MβCD for 1 h or with 50 mg/ml nystatin for 15 min. To enrich basal cholesterol, T-FGM cells were incubated with a solution of 2.5 mM MβCD plus 100 mM cholesterol for 16 h as described [57]. For long-term treatments, MβCD was used at 2.5 mM for 16 h.

### Measurements of intracellular vesicle mobility

T-FGM fibroblasts seeded in 13 mm glass coverslips were transfected with pEGFP-Cav-1 and observed 24 h later by living cell microscopy under controlled temperature and humidity. Images were obtained in a 1 mm section along the Z-axis every minute for a total of 15 min. A Z-axis composition for the time-dependent vesicle progression was made and their movement in up to 20 cells was followed by using the “Tracking” tool of the ImageJ software. Accumulated and Euclidean distances covered by each vesicle were calculated and compared essentially as described [16]. Results from the vesicles tracked from 3 different representative cells are shown.

### Fluorescence recovery after photobleaching (FRAP) measurements

T-FGM cells were transfected with pEGFP-Cav-1 and observed by confocal microscopy 24 h after transfection. Random cells were imaged and the fluorescence contained in a 10 μm-side region was bleached using 30 pulses of a 488 nm laser at 50% power (<50 mW). Images were taken before bleaching and every minute thereafter for a total of 15–20 min. Data were analyzed using the “Plot profiles” tool of the Image J software and FV10 software (Olympus).

### Endocytosis assays

Endocytosis measurements were done basically as described [58]. Briefly, T-FGM fibroblasts growing in complete medium were changed to serum-free medium for 2 h. During this time, cells were treated with MβCD, nystatin or cholesterol. After washing and addition of fresh serum-free medium, 1 μM BSA-FITC (Sigma) was added for 30 min or 24 h. Cell nuclei were stained with Hoechst 33342 for the last 20 minutes of incubation and cultures were washed and analyzed by confocal microscopy. Total fluorescence per cell was quantified in 3 different fields for each experimental condition.

### Statistical analyses

Data are shown as mean ± SD. Comparison between experimental conditions was done using GraphPad Prism 6.0 software (GraphPad). The student’s t test was used to analyze differences between two experimental groups or ANOVA for the analyses of three or more groups. Experiments were done in duplicate or triplicate in two or three biological replicates of each cell line.

## Additional file

Additional file 1: Figure S1. Negative control for Cav-1 immunofluorescence. T-FGM *AhR*+/+ fibroblasts were grown, fixed and processed for immunoflourescence using the same conditions as in Figure 1 except that the anti-Cav-1 antibody was not included. Secondary antibody used was Alexa 488. Cell nuclei were stained with DAPI. Transmitted light images were also taken from the same cultures. Bar corresponds to 50 μm.

## Abbreviations

AhR: Dioxin receptor
Cav-1: Caveolin-1, DRM, detergent resistant microdomains
FRAP: Fluorescence recovery after photobleaching
GFP: Green fluorescent protein
MbCD: Methyl-β-cyclodextin
TfR: Transferrin receptor
YFP: Yellow fluorescent protein
si-RNA: Small interfering RNA
