# Supplementary material for: The Dioxin receptor modulates Caveolin-1 mobilization during directional migration: role of cholesterol
Source: Cell Commun Signal. 2014 Sep 21;12:57. doi: 10.1186/s12964-014-0057-7 (PMC4172968; doi:10.1186/s12964-014-0057-7)
Supplement: Additional file 1: Figure S1. — Negative control for Cav-1 immunofluorescence. T-FGM AhR+/+ fibroblasts were grown, fixed and processed for immunoflourescence using the same conditions as in Figure 1 except that the anti-Cav-1 antibody was not included. Secondary antibody used was Alexa 488. Cell nuclei were stained with DAPI. Transmitted light images were also taken from the same cultures. Bar corresponds to 50 μm. [file 12964_2014_57_MOESM1_ESM.pptx]

## Slide 1
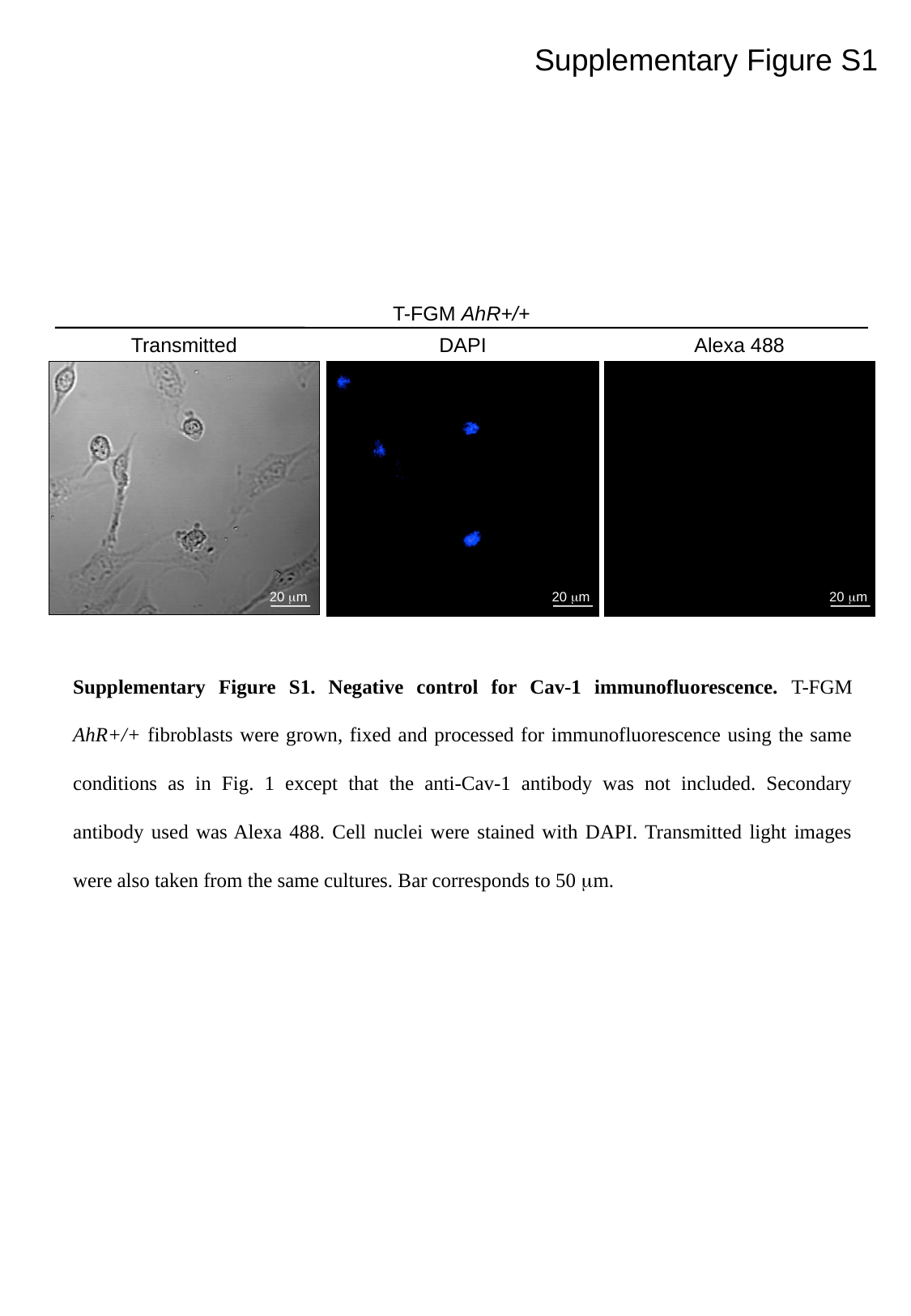

Supplementary Figure S1
T-FGM AhR+/+
Transmitted
DAPI
Alexa 488
20 mm
20 mm
20 mm
Supplementary Figure S1. Negative control for Cav-1 immunofluorescence. T-FGM AhR+/+ fibroblasts were grown, fixed and processed for immunofluorescence using the same conditions as in Fig. 1 except that the anti-Cav-1 antibody was not included. Secondary antibody used was Alexa 488. Cell nuclei were stained with DAPI. Transmitted light images were also taken from the same cultures. Bar corresponds to 50 mm.
